# Is palatal augmentation prosthesis effective in restoring speech, swallowing, and quality of life following cancer associated glossectomy? A systematic review

**DOI:** 10.1007/s00520-025-09241-y

**Published:** 2025-02-22

**Authors:** Ioli Ioanna Artopoulou, Xanthippi Dereka, Dimokritos Papalexopoulos, Maria Kouri, Nikolaos Nikitas Giannakopoulos

**Affiliations:** 1 Department of Prosthodontics, School of Dentistry, National and Kapodistrian University of Athens, Athens, Greece; 2 Department of Periodontology, School of Dentistry, National and Kapodistrian University of Athens, Athens, Greece; 3 Department of Oral Medicine & Pathology and Hospital Dentistry, School of Dentistry, National and Kapodistrian University of Athens, Athens, Greece

**Keywords:** Palatal augmentation prosthesis, Glossectomy, Speech, Swallowing, QoL

## Abstract

**Purpose:**

To evaluate the effect of palatal augmentation prosthesis (PAP) in improving speech intelligibility, swallowing efficacy, masticatory function, patients’ perception, and overall quality of life (QoL) in HNC survivors with partial or total glossectomy.

**Methods:**

The systematic review was conducted in compliance with the Preferred Reporting Items for Systematic Reviews and Meta-Analyses (PRISMA) guidelines and was registered in the International Prospective Register of Systematic Reviews (PROSPERO). The focused question was constructed according to the PICO (participant, intervention, comparison, and outcome) approach, and a three-stage screening in PubMed, Cochrane, Scopus, and Google Scholar databases with Medical Subject Heading (MeSH) terms was performed. The National Institute of Health (NIH) tool, “Quality Assessment Tool for Before-After (Pre-Post) Studies with No Control Group,” was employed for risk of bias assessment, and the data was synthesized qualitatively, according to the Synthesis without Meta-Analysis (SWiM) reporting guideline.

**Results:**

The initial search resulted in 131 articles, 80 were screened based on title and abstract, 11 full text articles were assessed for eligibility, and 9 articles were evaluated. Eight studies (88.9%) were characterized as fair and one as good. The total sample size included 176 participants. The “before” assessment was performed post-operatively, before PAP insertion, and the “after” at a time point ranging from after PAP insertion to a mean time of 9.3 (10.6) months, with a follow-up period ranging from 2 weeks to 82 months (mean time of 13.7 (17.3) months). Speech intelligibility was evaluated in eight studies. Six studies assessed swallowing and deglutition. Lingual movement dynamics of the center of the tongue, mastication ability, and patients’ reported experience with PAP were each evaluated in one study. QoL assessment was performed in two studies. The results of this systematic review indicate that PAP has a significant effect in improving speech intelligibility, swallowing efficacy, masticatory function, patients’ perceived outcomes, and overall QoL in HNC survivors with partial or total glossectomy.

**Conclusion:**

Due to the particular defect site–related disabilities, patients with tongue resection could benefit from PAP prostheses. However, future studies evaluating the role of PAP in alleviating speech and swallowing by means of current methods of assessment, as well as more explicit patient’s perceived treatment outcomes and QoL evaluations, are essential.

## Introduction

According to the National Cancer Institute’s Surveillance, Epidemiology, and End Results (SEER) Program, 58,540 people in the USA will receive a diagnosis of cancer of the oral cavity and pharynx in 2024, and unfortunately, about 19,230 people will die from the disease [1]. Oral tongue cancers will account for 19,360 estimated new cases, showing an increasing trend by 2.3% per year, whereas the estimated deaths will be 3320 [1, 2]. Following the National Comprehensive Cancer Network (NCCN) guidelines, surgical resection of primary oral tongue cancer, guided by the tumor location, depth of invasion, and imaging, with or without neck dissection, followed by adjuvant treatment (radiation and/or systemic therapy), remains the standard of care [3].

Surgical resections of oral tongue tumors may range from marginal resection to total glossectomy; cause functional and esthetic deformities, such as inefficient mastication, inadequate saliva control, dysphagia, distorted speech, and articulation deficits; and result in functional inconvenience, emotional distress, social isolation, and quality of life (QoL) impairment [4, 5]. The nature and the extent of the disability depend upon the location and size of the lesion, the post- chemo- and/or radiation therapy side effects, the amount of the resected tissues, and the method of surgical closure. Microvascular surgery offered more options in oral tongue reconstruction; however, the functional outcome remains varied, since free tissue transfers might be associated with bulky flaps, inadequate linguo-alveolar sulcus, the lack of voluntary control, limited or no sensation, and usually single directional movement, resulting in significant deglutition, speech, and articulation deficits [4, 6].

Alterations in oral tongue’s soft tissue bulk and contour, as well as decreased lingual mobility, due to possible tethering of the remaining structures or post-operative neurologic deficits, may result in severe dysfunction in bolus manipulation, oral preparatory and transit phases, leading to various degrees of swallowing difficulties. Adjuvant radiation therapy and the associated xerostomia may further impede deglutition, resulting in severe dysphagia and compromised QoL. Dysphagia may lead to serious life-threatening conditions including malnutrition, dehydration, aspiration induced pneumonia, and even death [7]. Furthermore, altered tongue volume, due to the head and neck cancer (HNC) ablative surgery or secondary to free flap reconstruction with restricted tongue or flap movement, can cause significant speech impairment [8].

Multidisciplinary management and team approach are essential in the restoration of oral tongue resection associated deficits. Maxillofacial prosthetic rehabilitation by means of a palatal augmentation prosthesis (PAP), followed by speech and swallowing therapy, is essential to aid deglutition and improve speech and articulation [9]. Prior to PAP fabrication, it is necessary that a thorough clinical examination, with appropriate diagnostic tests and radiographic study for swallowing assessment, should be completed to determine the risk of aspiration and confirm that maxillofacial prosthetic rehabilitation is appropriate. PAP is a removable intraoral maxillofacial prosthesis that alters the hard and/or soft palate topographical form adjacent to the tongue. It reshapes the hard palate, lowering the palatal vault prosthetically into the space of Donders, and allows for proper tongue/palate contact during speech and swallowing to compensate for impaired tongue mobility [4, 10–12].

Several studies have measured speech and swallowing parameters, quality of life and/or self-reported speech and dysphagia outcomes associated with partial or total glossectomy; however, no systematic review has been so far performed, in an effort to summarize the results of those primary studies in patients with HNC-associated tongue resection. The aim of this systematic review is to evaluate existing evidence on whether the use of PAP improves outcomes such as speech intelligibility, swallowing efficacy, patient’ perceived outcomes, and overall QoL in HNC patients who underwent partial or total glossectomy with or without surgical reconstruction.

## Methods

A systematic literature review was conducted in compliance with the Preferred Reporting Items for Systematic Reviews and Meta-Analyses (PRISMA) guidelines [13] and was registered in the International Prospective Register of Systematic Reviews (PROSPERO) as CRD42024554125.

### Study design and search strategy

The focused question of this review was constructed according to the PICO (participant, intervention, comparison, and outcome) approach [14] and was the following: “Is palatal augmentation prosthesis effective in restoring speech, mastication, and swallowing and improving QoL in patients following cancer associated glossectomy?”.

P (participants): patients with oral cancer-associated glossectomy, I (intervention): palatal augmentation prosthesis, C (comparison): non-treatment, O (outcome): speech, mastication, swallowing and QoL.

An extensive literature search was conducted by two independent reviewers (IIA and XD) in PubMed, Cochrane, Scopus, and Google Scholar databases for articles published until July 2024. The search strategy with Medical Subject Heading (MeSH) terms used for PubMed and keywords, along with the population, intervention, comparison, and outcome (PICO) format, are presented in Table 1. Moreover, issues of the following relevant journals were manually searched: *British Journal of Oral and Maxillofacial Surgery*, *Head & Neck*, *International Journal of Oral and Maxillofacial Surgery*, *Journal of Oral and Maxillofacial Surgery*, *Journal of Oral Rehabilitation*, *Journal of Prosthodontic Research*, *Journal of Prosthetic Dentistry*, *Supportive Care in Cancer*, and *Plastic and Reconstructive Surgery*. References of the included studies were also manually screened for additional eligible papers. No explicit search for grey literature was performed.

**Table 1:** PICO format justification and search terms

Population	Patients with partial or total glossectomy defects due to head and neck cancer ablative resection (± chemo-, radiation therapy) with or without surgical reconstruction
Intervention	Placement of a PAP following surgical resection
Comparison	Speech intelligibility Swallowing ability Lingual movement dynamics Masticatory function Patient reported outcomes Quality of life (QοL)
Outcome	Effect of PAP on speech, swallowing, tongue mobility, masticatory efficiency, patients’ perception and QoL
Search terms	((((((Palatal augmentation prosthe*[Title/Abstract]) OR (Prosth*rehabilitation[Title/Abstract])) OR (Prosth* restoration[Title/Abstract])) OR (Palatal speech aid [Title/Abstract])) OR (Palatal swallowing aid[Title/Abstract])) AND (Glossectomy[Title/Abstract])) OR (Tongue resection[Title/Abstract]) Filters: **English** Sort by: **Most Recent** (((“palatal augmentation prosthe*”[Title/Abstract] OR “prosth*rehabilitation”[Title/Abstract] OR “prosth* restoration”[Title/Abstract] OR ((“palatalization”[All Fields] OR “palatalized”[All Fields] OR “palatally”[All Fields] OR “palatals”[All Fields] OR “palate”[MeSH Terms] OR “palate”[All Fields] OR “Palatal”[All Fields] OR “palates”[All Fields]) AND “speech aid”[Title/Abstract]) OR ((“palatalization”[All Fields] OR “palatalized”[All Fields] OR “palatally”[All Fields] OR “palatals”[All Fields] OR “palate”[MeSH Terms] OR “palate”[All Fields] OR “Palatal”[All Fields] OR “palates”[All Fields]) AND “swallowing aid”[Title/Abstract])) AND “Glossectomy”[Title/Abstract]) OR “tongue resection”[Title/Abstract]) AND (english[Filter])

### Inclusion and exclusion criteria

A three-stage screening (titles, abstract, full text) was carried out in duplicate and independently by the two reviewers, according to preset eligibility criteria. Any disagreement was resolved by discussion, and, if necessary, a third reviewer (NNG) was consulted. The inclusion criteria concerned patients with cancer-associated glossectomy defects, (1) with or without surgical reconstruction of the defect, (2) who may have undergone other concurrent surgical procedures including the floor of the mouth and/or the mandible at the time of the glossectomy, (3) who may have received adjuvant chemo- or radiation therapy, (4) who may have received post-operative speech therapy, and (5) who were restored with a palatal augmentation prosthesis. Only completed clinical trials, randomized controlled (RCTs) or non-RCTs, cross-sectional, and cohort studies that involved *N* > 5 patients and were published in English language were included. Studies reporting interventions on participants with non-cancer-related glossectomies, tongue mobility deficits or non-glossectomy-related dysphagia, and studies with tongue prostheses placed in the mandible were excluded. Case reports, case series, reviews, and review protocols were also excluded.

### Risk of bias assessment

The National Institute of Health (NIH) tool, “Quality Assessment Tool for Before-After (Pre-Post) Studies with No Control Group,” [15] was employed. This instrument, according to the search strategy, is most suitable for the type of studies we expect to include. The level of agreement between the reviewers for the second and third stage of screening, as well as the quality assessment, was calculated using kappa statistics. Two calibrated reviewers (IIA and NNG) assessed the quality of the included studies independently. All disagreements were solved by reaching consensus after discussion between the reviewers.

### Data extraction

The full text of all possibly eligible studies was sought for, and after confirming eligibility, detailed information was extracted in prepared electronic data extraction forms, in duplicate, and was cross-checked for accuracy. The following information will be extracted from the full text of the included articles: first author name, title, year of publication, type of study, number of patients, type of surgical resection, type of prosthetic intervention, and measures of function. In case of missing data, the authors of the papers were contacted.

### Data synthesis

Due to the heterogeneity of the included studies, in study design, time and duration of the intervention, the reported outcome measures, and the reported methods of outcome evaluation, conducting a meta-analysis of the effect estimates was not considered possible. Therefore, the data was synthesized qualitatively, according to the Synthesis without Meta-Analysis (SWiM) reporting guideline [16]. In the synthesis, studies were grouped by review outcomes and the results were synthesized for each expected outcome (speech intelligibility, swallowing ability efficacy, lingual movement dynamics, masticatory function, patient reported outcomes, and QoL) when at least three studies (with different patient samples) reported on them. The data synthesis occurred individually for each expected outcome, and we also tried to account for the time frame. Hence, the summary effect measures were not determined in advance but were adapted according to the number of studies reported on each outcome.

## Results

### Study selection

**Fig. 1:**
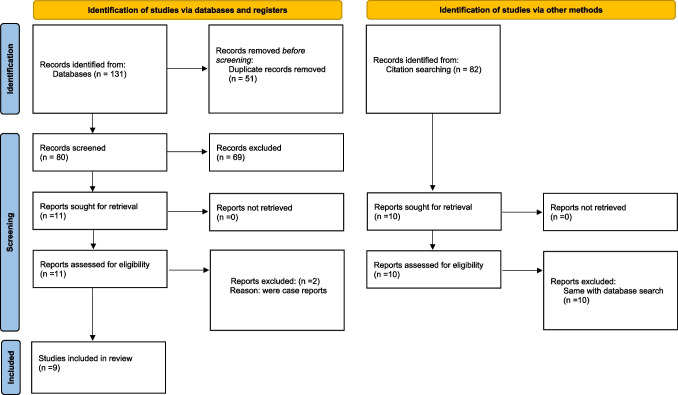
The PRISMA flow chart

Figure 1 shows the selection procedure of the studies as a flow chart. The initial search resulted in 131 articles. After duplicate removal, 80 were screened based on title and abstract, and 69 were not relevant for the purpose of this review; consequently, they were rejected. Eleven full-text articles were assessed for eligibility. Two manuscripts [17, 18] were excluded because they were case reports, and a total of 9 articles were included in this review. Seven studies were retrospective clinical trials with no separate control group [9, 11, 12, 19–22] and two retrospective cross-sectional clinical trials [23, 24]. The quality of the selected studies was assessed in duplicate and independently with the NIH Quality Assessment Tool for Before-After (Pre-Post) Studies with No Control Group, and the results are presented in Table 2. Eight studies [19, 20, 22–24] (88.9%) were characterized as fair and one [21] as good. The criteria mostly associated with risk of bias were the lack of report of blinding, the lack of report of multiple measuring, and the small sample size. The majority of the studies were conducted in the USA, two in Japan, one in Brazil, one in Italy, and one in Sweden. In general, the sample size of the studies was rather small, with a total of 176 participants; more than half (5/9, 55.6%) of the studies had ten or less participants. The first assessment of the participants was performed post-operatively, before PAP insertion, and then a follow-up occurred at certain time points ranging from immediately after the PAP insertion to a mean time of 9.3 (10.6) months, with the follow-up period ranging from 2 weeks to 82 months (mean time of 13.7 (17.3) months). The percentage of tongue resection was reported in 8 out of 9 studies (166 participants); 42 participants (42/166, 25.3%) had a total glossectomy, 8 (8/166, 4.8%) a subtotal glossectomy, and the rest (116/160, 69%) had ≤ ¾ of the tongue resected, resulting in restriction of tongue mobility which is reported only in 2 studies. The key characteristics of each included study are provided in Table 3 with more detail.

**Table 2:**
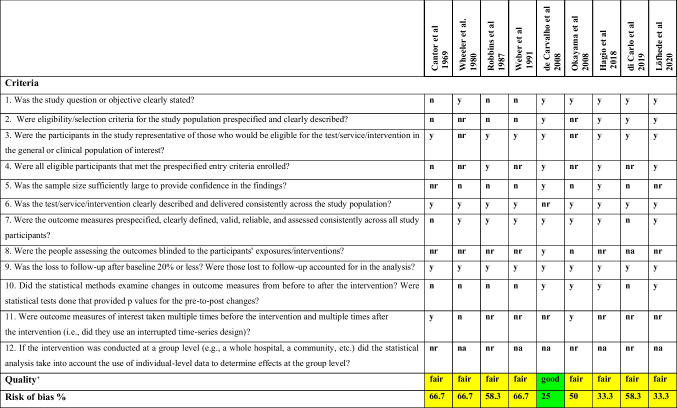
Results of risk of bias assessment of the included studies with the NIH Quality Assessment Tool for Before-After (Pre-Post) Studies with No Control Group

*y*, yes; *n*, no; *nr*, not reported; *na*, not applicable

^**+**^Quality is rated as good (< 25% risk of bias), fair (26–74% risk of bias), and poor (> 75% risk of bias)

**Table 3:** Study characteristics of the included studies

Study	Location	Study design	Age range (average)	Patients *N* (male/female)	Comparison	Follow-up		Surgical reconstruction
Cantor et al. 1969	USA	Retrospective non-RCT	48–75 (NR)	10 (NR)	-Baseline: post-op, before PAP insertion −2 weeks post-insertion of PAP	2 weeks		Primary closure (*n* = 10)
Wheeler et al. 1980	USA	Retrospective non-RCT	46–74 (NR)	10 (NR)	-Baseline: post-op, before PAP insertion −4–6 weeks post-insertion of PAP	4–8 weeks		Primary closure (*n* = 6) Tongue flap (*n* = 3) Titanium basket implant (*n* = 2)
Robbins et al. 1987	USA	Retrospective non-RCT	45–64 (55.4)	10 (7/3)	-Baseline: 2 weeks post op -Post-XRT -Post-insertion of PAP −3 months post-insertion of PAP −6 months post-insertion of PAP	6 months		Pectoral myocutaneous flap (*n* = 2) Skin graft (*n* = 1)
Weber et al. 1991	USA	Retrospective non-RCT	33–78 (58)	27 (18/9)	-Baseline: post-op (*n* = 27) -Following PAP insertion (*n* = 18)	14 months		Pectoral myocutaneous flap (*n* = 23) Lateral trapezius flap (*n* = 1) Skin graft (*n* = 1) Infrahyoid myocutaneous flap (*n* = 1) Primary closure (*n* = 1)
Okayama et al. 2008	Japan	Retrospective non-RCT	48–78 (65)	7 (5/2)	-Baseline: post-op -Following PAP insertion	30–82 months		Forearm flap (*n* = 3) Latissimus dorsi/rib flap (*n* = 1) Scapular flap (*n* = 1)
de Carvahlo-Teles et al. 2008	Brazil	Retrospective non-RCT	30–80 (55)	36 (33/3)	-Baseline: 3 months post-op after speech therapy and before PAP insertion -After using PAP for a mean time of 9.3 months	3–41 months		Pectoralis flap (*n* = 34) Platysma myocutaneous flap (*n* = 2)
Hagio et al. 2018	Japan	Retrospective non-RCT	NR (72.4)	50 (34/16)	-Baseline: post-op, before insertion of maxillofacial prosthesis (*n* = 50) - 1 month following PAP insertion (*n* = 5)	NR		Myocutaneous reconstruction (*n* = 3)
Di Carlo et al. 2019	Italy	Retrospective cross-sectional	67–75 (72)	6 (4/2)	- Baseline: post-op, 1 week before PAP insertion - 2 months following PAP usage	2 months		NR
Löfhede et al. 2020	Sweden	Retrospective cross-sectional	47–81 (63.2)	20 (11/9)	-Baseline: post-op, before PAP insertion - Min. 1 month following PAP usage	NR		Fibula transplant (*n* = 1) Pectoralis flap (*n* = 1) Radicalis flap (*n* = 1)
Study	Resection to adjacent structures	Adjuvant treatment	Evaluation of post-operative restriction of tongue mobility	% of tongue resected	Post-op speech and swallowing therapy before PAP insertion	Time of PAP insertion		Complications
Cantor et al. 1969	NR	NR	Moderate restriction (*n* = 5) Extreme restriction (*n* = 5)	NR	NR	2 weeks post-op		NR
Wheeler et al. 1980	Anterior FOM (*n* = 3) SL (*n* = 1) Tonsil, BOT (*n* = 6) Anterior mandible (*n* = 3) Mandible (*n* = 6) Radical ND (*n* = 10)	NR	NR	10% (*n* = 4) 20% (*n* = 2) 50% (*n* = 2) 80% (*n* = 1) 90% (*n* = 1)	2–6 months	2–6 months post-op		NR
Robbins et al. 1987	BOT (*n* = 5) Pharynx (*n* = 1) Modified ND (*n* = 9) Mandible (*n* = 2) Larynx (*n* = 1) Laryngeal suspension (*n* = 1) FOM (*n* = 3)	Post-op XRT (*n* = 5)	Elevation: 0% (*n* = 2) 25% (*n* = 1) 25–50% (*n* = 1) 50% (*n* = 1) 50–75% (*n* = 3) 50–100% (*n* = 1) Lateralization: 0% (*n* = 3) 25% (*n* = 1) 25–75% (*n* = 2) 50% (*n* = 1) 0–75% (*n* = 2) 25–100% (*n* = 1) Elongation: 0% (*n* = 5) 25% (*n* = 2) 50% (*n* = 2) 75% (*n* = 1)	0% (*n* = 1) 25% (*n* = 1) 25–50% (*n* = 3) 50% (*n* = 1) 50–75% (*n* = 1) 75% (*n* = 1) 100% (*n* = 2)	NR	Post-XRT		NR
Study	Resection to adjacent structures	Adjuvant treatment	Evaluation of post-operative restriction of tongue mobility	% of tongue resected	Post-op speech and swallowing therapy before PAP insertion		Time of PAP insertion	Complications
Weber et al. 1991	Laryngeal suspension (*n* = 18)	Post-op XRT (*n* = 18)	NR	≥ ¾ of tongue (*n* = 13) total glossectomy (*n* = 14)	NR		post-op	Wound infections (*n* = 4) Partial loss of skin portion of myocutaneous flap (*n* = 7)
Okayama et al. 2008	Mandible (*n* = 3) FOM (*n* = 1) ND (*n* = 7)	NR	NR	Partial resection (*n* = 3) Hemiresection (*n* = 3) Subtotal resection (*n* = 1)	NR		Post-op	NR
de Carvahlo-Teles et al. 2008	Mandible (*n* = 12)	Post-op XRT (*n* = 36) Post-op chemo (*n* = 2)	NR	Hemiglossectomy (*n* = 6) Subtotal glossectomy (*n* = 4) Total glossectomy (*n* = 26)	3 months		22.3 months post-op	NR
Hagio et al. 2018	FOM (*n* = 3)	NR	NR	Hemiglossectomy (*n* = 2) Oral floor (*n* = 3)	NR		NR	NR
Di Carlo et al. 2019	Mandible (*n* = 1) Pharynx (*n* = 2) FOM (*n* = 2)	Post-op XRT (*n* = 1) Post-op chemo (*n* = 1) Post-op XRT/chemo (*n* = 2)	NR	Hemiglossectomy (*n* = 6)	NR		NR	NR
Löfhede et al. 2020	BOT (*n* = 2) Pharynx (*n* = 1) Radical ND (*n* = 5) Mandible (*n* = 9) Larynx (*n* = 1) Tonsil (*n* = 3) FOM (*n* = 3)	Post-op XRT (*n* = 5) Post-op XRT/brachy (*n* = 14)	NR	Partial resection (*n* = 4) Hemiresection (*n* = 4)	NR		Min. 1 month post-op	NR
Study	Evaluation of speech	Evaluation of swallowing/deglutition	Assessment of lingual movement dynamics of the center of the tongue	Patient- reported experience	Masticatory function		QoL	
Cantor et al. 1969	Five speech pathologists K, G sounds in ten words	NR	NR	NR	NR		NR	
Wheeler et al. 1980	(i) 5 untrained listeners conversational speech (ii) Videofluoroscopic evaluation lateral plane: 9 single-syllable CVC words requiring tongue-palate contact or approximation A-P plane: 6 single-syllable CVC words with /s/ and /sh/	Videofluoroscopic evaluation 3 swallows of 3 materials: liquid, thin paste, thick paste No head stabilization	NR	NR	NR		NR	
Robbins et al. 1987	One speech pathologist - “Rainbow passage” - conversational speech - words with target sounds Articulation scale	One speech pathologist - Clinical examination of swallowing - Evaluation of grosser motor movement of swallowing (swallowing speed, head movements, bolus pooling, aspiration) Deglutition scale	NR	NR	NR		NR	
Weber et al. 1991	One speech pathologist Assessment of speech intelligibility	Videofluoroscopic evaluation: - 2–3 ml to assess aspiration - 5–10 ml for complete feeding assessment	NR	NR	NR		NR	
Okayama et al. 2008	NR	Repetitive saliva swallowing test (RSST) - Dry swallows for 30 s - Total time for 3 swallows	Ultrasound imaging Swallowing to evaluate: - Grooving depth - Grooving duration - Duration of contact with palate - Total duration of lingual movement during swallowing	NR	NR		NR	
Study	Evaluation of speech	Evaluation of swallowing/deglutition	Assessment of lingual movement dynamics of the center of the tongue	Patient-reported experience	Masticatory function	QoL		
de Carvahlo-Teles et al. 2008	Two speech pathologists - 30-s spontaneous speech - Repetition of 18 syllables with plosive, fricative voiced and voiceless sounds (nasal, liquid sounds together with the vowel /a/ - Sustained emission of the vowels /a,e,é,i,o,ó,u/ - Spectrographic assessment of the formants of the seven vowels of Brazilian Portuguese	NR	NR	NR	NR	NR		
Hagio et al. 2018	Speech intelligibility test (25 monosyllables: /kш/, ke/, /ta/, /te/, /to/, /лi/, /çi/, /mi/, /ri/, /rш/, /re/, /ge/, /go/, /da/, /de/, /do/, /bi/, /kja/, /kjo/, /∫a/, /∫ш/, /t/∫a/, /t/∫o/, /dзa/, /dзш/)	Swallowing ability score	NR	NR	Sato method assessed masticatory ability	OHIP-J54		
Di Carlo et al. 2019	Speech therapist Phonetic test (56 words)	Pharyngeal swallowing pressure test	NR	NR	NR	- VAS - OHIP-14		
Löfhede et al. 2020	Three speech pathologists - Audiovisual recordings - Swedish dysarthria test - Additional sentences with velar sounds -*n* = 19/20 evaluated participants	NR	NR	- Speech - Drooling, oral transport - Frequency of using PAP -*n* = 20/20 evaluated participants	NR	NR		

*PAP* palatal augmentation prosthesis, *non-RCT* non-randomized clinical trial, *post-op* post-operative, *XRT* external beam radiation therapy, *brachy* brachytherapy, *FOM* floor of the mouth, *SL* supraglottic laryngectomy, *BOT* base of tongue, *ND* neck dissection, *NR* not reported, *CVC* consonant-vowel-consonant, *A-P* anteroposterior

### Speech intelligibility evaluation

In six studies (*N* = 109, 61.9%) [9, 11, 12, 21, 23, 24], speech evaluation was performed by speech pathologists. Hagio et al. (*N* = 50, 28.4%) [22] did not report whether the assessment was assisted by a speech specialist; one study (*N* = 1, 5.7%) [19] indicated speech assessment by untrained listeners, and Okayama et al. (*N* = 7, 4%) [20] did not evaluate the speech following PAP insertion. In three studies (*N* = 66, 37.5%) [19, 21, 24], speech intelligibility assessment was performed by means of conversational speech, whereas in seven studies (*N* = 159, 90.3%) [9, 11, 12, 21–24], words with targeted sounds and specifically formulated passages were used. Additionally, Wheeler et al. [19] and de Carvahlo-Teles et al. (*N* = 46, 26.1%) [21] employed videofluoroscopy and spectrographic assessment, respectively, for further speech evaluation.

### Evaluation of swallowing-deglutition

Only six studies evaluated swallowing and deglutition of the participating patients (*N* = 95, 54%) [9, 11, 19, 20, 22, 23]. Two studies conducted videofluoroscopic evaluation (*N* = 37, 21%) [12, 19]. One study utilized the swallowing ability test (*N* = 50, 28.4%) [22], another study the pharyngeal swallowing pressure test (*N* = 6, 3.4%) [23], and a following one performed the repetitive saliva swallowing (RSS) test (*N* = 7, 4%) [20]. Finally, in one study, a trained speech pathologist made a thorough clinical examination and evaluated the grosser motor movement of swallowing (*N* = 10, 5.7%) [11].

### Additional assessments

The lingual movement dynamics of the center of the tongue was assessed by one study, using ultrasound imaging to evaluate the grooving depth, the grooving duration, the duration of contact with palate, and the total duration of lingual movement during swallowing (*N* = 7, 4%) [20]. Patients’ perceptions regarding their experience, such as frequency of PAP use, speech, drooling, and oral transport, were documented by the investigators of one study (*N* = 20, 11.4%) [24], while the Sato method of chairside scoring of chewing function was used for evaluating the masticatory ability of the participants in another study (*N* = 50, 28.4%) [22]. QoL assessment was only performed in two studies (*N* = 56, 31.8%) [22, 23], using the Oral Health Impact Profile (OHIP)-J54 and OHIP-14 questionnaires, along with the Visual Analogue Scale (VAS).

### Study outcomes

The results of the included studies are presented cumulatively in Table 4. Significant speech improvement was observed in five studies [11, 12, 19, 21, 23], whereas Cantor et al. [9] indicated significant speech improvement only in patients with severe tongue post-surgical restriction. Hagio et al. [22] reported no significant difference in speech with or without PAP. In the study by Löfhede et al. [24] in almost half of the patients, speech amelioration following PAP insertion was observed, three participants were better without the prosthesis, and in seven patients, no significant differences, before and after the use of PAP, were reported. Swallowing ability was improved in all the studies assessing deglutition [11, 12, 19, 22, 23], with a significant decrease in oral preparatory/transit and pharyngeal transit time, whereas following PAP insertion mean pharyngeal swallowing pressure was significantly greater. The only exception was the study by Okayama et al. [20] where there was no significant improvement in swallowing while using the PAP. Evaluation of lingual movement dynamics of the center of the tongue showed a decrease in the tongue contact with palate duration, along with a decreased total duration of lingual movement during swallowing [20]. Regarding the patients’ reported outcomes, Löfhede et al. [24] indicated that following PAP use, *n* = 12 (63%) patients had an easier or better speech experience, *n* = 2 (10.5%) patients reported that some sounds were better and some worse), and *n* = 2 (10.5%) patients described a worse speech experience. Regarding oral transport of food and drink, *n* = 7 (35%) participants reported benefits of the PAP for oral transport and use at mealtimes, whereas *n* = 10 (50%) patients reported that the device was taken out during mealtimes and that they did not benefit from the device in terms of oral transport. Furthermore, *n* = 7 (35%) reported less drooling because of easier oral transport of saliva to the pharynx, whereas three patients (15%) did not attend the follow-up. Patients’ experiences regarding usage of the PAP indicated that *n* = 9 (45%) used the device all day, *n* = 7 (35%) used it part of the day, and *n* = 4 (20%) did not use the prosthesis at all. Only one study by Hagio et al. [22] evaluated masticatory function by means of patient’s perceived masticatory ability. The results indicated that PAP did not further improve the masticatory ability of the participants. In both studies [22, 23], QoL appeared to be significantly improved following the PAP insertion, in all participants.

**Table 4:** Results of the included studies

Study	Speech intelligibility	Swallowing	Lingual movement dynamics of the center of the tongue	Patient-reported experience	Mastication function		QoL
Cantor et al. 1969	Patients with severe tongue restriction (*n* = 5): significant improvement in speech intelligibility (+ 13.8 to + 36.0) with PAP Patients with moderate tongue restriction (*n* = 5): consistent decrease in speech intelligibility (− 1.6 to − 10.6) with PAP	NR	NR	NR	NR		NR
Wheeler et al. 1980	Improvement in speech intelligibility (*N* = 10) with PAP Mean improvement = 12.5% (9–18%) Speech understandability > 90% (*n* = 6)	Oral transit time decreased with PAP: Liquid: Mean reduction 1.29 s (*n* = 10) Thin paste: Mean reduction 2 s (*n* = 10) Thick paste: Mean reduction 1.35 s (*n* = 10) Pharyngeal transit time decreased with PAP: Liquid: Mean reduction 0.8 s (*n* = 5) Thin paste: Mean reduction 1.2 s (*n* = 5) Thick paste: Mean reduction 1.2 s (*n* = 5)	NR	NR	NR		NR
Robbins et al. 1987	Immediate improvement: 4.5 points with PAP Long-term improvement: 3.4 points with PAP	Oral preparatory-oral phases: Immediate improvement: 3.5 points with PAP Long-term improvement: 2.2 points with PAP	NR	NR	NR		NR
Weber et al. 1991	With PAP (*n* = 18) - Good speech quality (*n* = 7) - Fair speech quality (*n* = 11)	With PAP (*n* = 18) - Accomplished deglutition and oral alimentation (*n* = 13) - Not (*n* = 5)	NR	NR	NR		NR
Okayama et al. 2008	NR	No significant improvement in swallowing frequency during 30 s, nor in the total duration of 3 swallows with PAP	- Duration of lingual contact with palate was decreased (621.8 ± 364.9 ms) with PAP - Total duration of lingual movement during swallowing was decreased (1245.6 ± 272.5 ms) with PAP	NR	NR		NR
de Carvahlo-Teles et al. 2008	- Improvement in intelligibility of spontaneous speech with PAP - Improvement in intelligibility of syllables with PAP - Increased F2 and F3 values for all vowels with PAP - Increased F1 values for the vowels /o,ó,u/ with PAP - The values of many vowel formants were closer to normal intelligibility with PAP	NR	NR	NR	NR		NR
Hagio et al. 2018	No significance difference with or without PAP	Swallowing ability was improved with PAP	NR	NR	PAP did not further improve the masticatory ability		Improved with PAP
Di Carlo et al. 2019	Improved speech with PAP	Mean pharyngeal swallowing pressure was significantly greater with PAP	NR	NR		NR	Improved with PAP
Löfhede et al. 2020	Better with PAP (*n* = 9) Better without PAP (*n* = 3) No significant difference with or without PAP (*n* = 7)	NR	NR	**a) Speech experience:** - Easier or better (*n* = 12/19), some sounds better and some worse (*n* = 2/19), worse (*n* = 2/19) **b) Experience with oral transport of food or drink and drooling:** - Better (*n* = 7/20), no benefit during meals (*n* = 10/20), did not attend the follow-up (*n* = 3/20) - Easier to transport the saliva, back to the pharynx or forward to spit (*n* = 7/20), no benefit (*n* = 7/20), did not attend the follow-up (n = 3/20) **c) Frequency of using the device** - All day (*n* = 9/20), part of the day (*n* = 7/20), not at all (*n* = 4/20)		NR	NR

*PAP* palatal augmentation prosthesis, *NR* not reported

## Discussion

The aim of this systematic review was to present the available literature and describe the role of PAP in improving speech intelligibility, swallowing efficacy, masticatory function, patient’s perceived outcomes, and overall QoL in HNC survivors with partial or total glossectomy. Cancers that involve the oral tongue and their associate treatment have potentially serious functional and esthetic consequences, associated with sensory motor deficits and loss of important anatomic structures with varying degrees of disability. The resultant functional impairments, due to the inadequate oral tongue volume and/or restricted tongue mobility, include altered speech intelligibility, incompetent mastication, and swallowing symptoms or dysphagia. The results of this systematic review indicate that treatment with PAP could significantly alleviate symptoms associated with defective speech and swallowing, decrease both the duration of lingual movement and lingual-palate contact during swallowing and improve QoL. Regarding its methodology, the systematic review followed the PRISMA [13] and the SWiM guidelines. [15] The included studies were retrospective; therefore, the confidence of the results may be reduced. The study design “before-after (pre-post) with no separate control group,” followed by all included studies, may be convenient for the specific population and studies’ settings but may increase the overall risk of bias, especially for the more subjective outcomes.

In all the included studies [9, 11, 12, 19–24], speech evaluation was mainly based on perceptual assessments, as speech-language pathologists and/or untrained listeners rated the quality of patients’ speech production in conversational speech and/or targeted sounds. However, these methods have certain limitations, since they usually show great subjectivity, are based on comparisons with another voice or with the listener’s previous impressions of the same voice, their reliability is mostly listener-depended, and often present inter-judge and intra-judge variability [8, 25]. If the rater is an untrained listener, commonly familiar with the patient, the results can be very different and perhaps unreliable when compared to an expert evaluator, whereas most of these tools were developed for patients with laryngectomy and were not originally intended for patients following oral tongue resection [8]. Recently, technological advancement allowed the development of new tools and more reliable methods of speech assessment based on objective data. These include acoustic analyses, the Assessment of Intelligibility of Dysarthric Speech (AIDS) tool, and patient reported self-perceived functional outcomes, such as the Speech Handicap Index (SHI) [8, 26, 27].

With one exception [20], all the included studies evaluating deglutition [9, 11, 19, 22, 23] indicated that most participants’ swallowing ability was improved following PAP insertion, with significant reduction in oral and pharyngeal transit times. The assessment was performed with the following methods: Videofluorcopic Swallow Study (VFSS) [12, 19], with or without modified barium swallow, swallowing ability score measurements [22], pharyngeal swallowing pressure test [23], Repetitive Saliva Swallowing (RSS) test [20], and clinical examination followed by grosser motor movement of swallowing [11]. Disruption of unrestricted swallowing and the relevant symptomatology results in dysphagia that could either present at the time of the diagnosis, due to tumor involvement in the upper aerodigestive structures responsible for normal swallowing, or occur as post-treatment morbidity due to surgery or adjuvant treatment modalities. According to the most recent experts’ consensus statement on the evaluation and management of dysphagia in HNC patients, the timing of dysphagia screening is crucial, since it is best performed prior to HNC therapy [7], a parameter that was not followed in the included studies, since the evaluation of deglutition was always performed post-operatively. The inclusion of nutrition screening using validated screening tools and patient specific characteristics, as well as speech-language pathologists’ swallow assessment, should also be included and initiate before cancer therapeutic interventions [7]. Patients who fail the pre-treatment dysphagia screening should be referred for further pre-treatment instrumental swallow evaluation, by means of Flexible Endoscopic Evaluation (FEE) or VFSS; the later method was utilized in two of the included studies [12, 19] and established baseline swallowing function and impairment in HNC patients who report symptoms of dysphagia or who are at risk of developing dysphagia during treatment. [7]. During HNC treatment, obtaining patient reported dysphagia-related outcomes by means of Eating Assessment Tool (EAT) and/or MD Anderson Dysphagia Inventory (MDADI) is essential for monitoring treatment-related morbidity [7].

Speech and swallowing functions following glossectomy correlate with the mobility of the remaining oral tongue and/or reconstruction flap structures. Following resection, the dorsal surface of the remaining or reconstructed oral tongue should be able to accomplish to some extent contact with the palate, achieve proper phonation and articulation, and facilitate adequate swallowing by squeezing and collecting the bolus [27]. Only one of the included studies evaluated the lingual mobility and indicated that following PAP insertion, there was shorter duration of the lingual-palatal contact, as well as decreased duration of the total tongue movement during swallowing [20]. The results of this study indicate that lowering the palatal surface, by placing the PAP, the distance between the tongue and the palate is shortened and the time required by the tongue to achieve palatal contact during swallowing is decreased. In addition, one study evaluated the mastication ability following PAP insertion, employing the Sato method [28] which appeared to be unaltered. With the selected method, chairside scoring data is collected by means of a questionnaire that evaluates the patients’ perceptions of masticatory ability. It is a subjective method, initially designed for complete denture wearers and cannot determine masticatory performance or degree of comminution [29]. However, it should be noted that unless replacing missing teeth, the specific prosthesis is not usually fabricated for that cause.

Although subjective, cancer survivors’ reported experience regarding treatment and rehabilitation should be carefully considered. The correlation of clinical objective and patient perceived measures will give a more thorough understanding regarding the degree of impairment, as well as the success of the rehabilitative outcome. In the included study [24], which evaluated the patients’ experience, self-reported experiences were recorded after using the prostheses, but no specific question protocol or validated questionnaires were used. Furthermore, the clinical value of the QoL questionnaires’ scores is difficult to interpret, since there were significant differences between the involved participants. In one study, all patients received myocutaneous reconstruction [22], whereas in another study, no information regarding the surgical reconstruction was reported [23]. In addition, information regarding post-operative adjuvant treatment was not available for both included studies.

One limitation of this systematic review is the lack of synthesis of the results, due to the small number of studies reporting each outcome. Moreover, the large heterogeneity of the study samples, treatment protocols, assessment methods, and observation time make a numerical synthesis of the results meaningless. Hence, the collected data does not allow for robust conclusions regarding the effects of PAP on the studied outcomes. Further methodological concerns are related to the size of the defect and the extent of the surgical resection to the adjacent oral structures. Baseline “before” time ranged from immediately post-operatively to 3 months post-operatively following pre-prosthetic speech therapy, whereas the “after” time ranged from immediately after PAP insertion to 9.3 months after PAP delivery. The structures that surround the tongue including the floor of the mouth, the mandible, and the base of the tongue along with the tonsillar area significantly assist the oral tongue function. Several studies indicated that there are significant functional and QoL differences in patients with glossectomy, associated with the defect size and the extension of the surgical resection to the adjacent tissues regardless potential reconstructive surgery [30, 31].

Current clinical practice guidelines in head and neck oncology focus on organ preservation treatment protocols; however, this is not necessarily translated to organ function preservation. Because of the particular defect site–related disabilities, patients with tongue resection bear social presentation, social communication, and self-image challenges, as well as psychosocial morbidity and QoL burdens. Assessment of treatment outcomes should include not only survival rates but also specific measures associated with rehabilitative approaches that reflect patient well-being. Future studies are essential to guide clinical care and patient counselling on expected short- and long-term functional and QOL outcomes.

## Conclusions

HNC survivors with partial or total glossectomy could benefit from PAP. Post-operative treatment with PAP could significantly alleviate symptoms associated with defective speech and swallowing, decrease the duration of lingual movement and lingual-palate contact during swallowing, and improve QoL. Further evaluation of the role of PAP in alleviating speech and swallowing by means of current methods of assessment, as well as patient’s perceived treatment outcomes, adaptation, and QoL measures, is essential.

## Data Availability

The data is available at the NKUA.

## Code availability

The statistical package used is licensed at NKUA.
